# Influence of patella height after patella fracture on clinical outcome: a 13-year period

**DOI:** 10.1007/s00402-021-03871-7

**Published:** 2021-04-06

**Authors:** Pesch Sebastian, Zyskowski Michael, Greve Frederik, Müller Michael, Wurm Marcus, Crönlein Moritz, Biberthaler Peter, Kirchhoff Chlodwig

**Affiliations:** Department of Trauma Surgery, Klinikum Rechts Der Isar, Technical University of Munich, Ismaninger Strasse 22, 81675 Munich, Germany

**Keywords:** Patella fracture, Comminuted patella fracture, Patella alta, Patella baja, Locking plate fixation, Patient-oriented outcome measurement

## Abstract

**Introduction:**

The incidence of patella fracture is statistically low (0.5–1.5%) compared to other fractures of the extremities [Patella fractures 76(10):987–997, 2005]. In the latter research, patella fractures if treated surgically present an overall inferior functional outcome. Little is known about the influence of the postoperative patella height on the clinical outcome. Therefore, the aim of our study was to analyse the influence of the patella height on the patients’ functional outcome after surgery.

**Methods:**

In this retrospective study the in-house trauma register of our level I University trauma center was screened for patients suffering patella fractures treated surgically. Patella height of the same patients was evaluated on lateral X-rays using the Insall–Salvati Ratio (ISR). The patients’ X-rays were analyzed at two time points for the ISR, whereas group A presents ISR data right after surgery and group B data at the latest follow up (minimum 6 weeks). The change of mean ISR at both time points was tested for significance. The functional outcome was measured by the “Munich Knee Questionaire” (MKQ). These MKQ results of different patella heights and fracture types were compared.

**Results:**

The screening of our in-house trauma register revealed 375 patients between the years 2003 and 2016. Out of these 54 patients (34f, 20 m) were enrolled. In detail the follow-up time for ISR between group A and B accounted for a mean of 503.8 ± 655.7 days. The MKQ was assessed at a mean of 1367.0 ± 1042.8 days after surgery. According to the AO-classification 10% AO.34 type B and 90% AO.34 type C fractures were found. Group A showed in 9.1% a patella baja and in 27.3% a patella alta compared to group B presenting 20.0% patella baja and 14.5% patella alta. There was no significant difference in functional outcome referring to the MKQ in patella alta (MKQ 69.0% ± 18.2) or baja (MKQ 67.1% ± 17.9) (*p* = 0.9). No significant functional difference between AO34.type B (MKQ 74.5% ± 11.0) and AO34.type C fractures (MKQ 64.0% ± 15.0) resulted (*p* = 0.1).

**Conclusion:**

Our results demonstrate that different postoperative patella heights apparently do not influence the functional outcome in the short follow-up.

## Introduction

Incidence of patella fractures is statistically low (≤ 1%) when compared to [1] other extremity fractures, with a peak incidence in the 3rd and 6th decade of life [2]. Statistically male individuals suffer from patella fractures twice as much as women. Mostly a direct blunt trauma to the anterior surface of the patella causes transverse, longitudinal or comminuted fractures. Injuries to the patella resulting from acute extending movements in a flexed knee position are rare. Besides restoring the compromised functional knee-extending complex, indications for surgical treatment include a fracture gap of 2–3 mm and joint surface incongruence possibly resulting in posttraumatic osteoarthritis [3]. Transverse fractures of the patella encountering younger patients are mostly treated by surgery performing open reduction and internal fixation [4, 5]. Tension band wiring with its different modifications (K-wires, figure-of-eight wiring, equatorial-cerclage) are well established in the surgical community [6]. Locking plate fixation apparently shows better biomechanical results in stress-loading tests in models and cadaver studies [6, 7]. Predictors for a good clinical outcome are a precise reconstruction of the joint surface, superior retention and finally healing of the fracture [8]. In the current literature, less than 50% of the patients present with good functional outcome following operative treatment [9, 10]. Knowing the importance of the patella height for joint motion, there is a surprising lack of data of patella height on the postoperative clinical outcome following patella fracture. The low clinical and functional outcome after patella fractures is discussed in either biological aspects (pathologies of the synovia and/or Hoffa’s fat pat) or referring in to biomechanical malformations due to, e.g., increased patella height with raised patella-femoral contact pressure during extensive movements [11–13]. Therefore, the aim of our study was to retrospectively analyze the influence of the postoperative patella height on the functional outcome following operatively treated patella fractures over a 13-year period.

## Materials and methods

Between 2003 and 2016 a retrospective data analysis was conducted regarding patella fractures at our University level-I trauma center. Inclusion criteria were age between 18 and 85 years, patella fractures according to the AO classification, no concomitant knee injury and surgical treatment.

In general, the surgical procedure was adapted to the fracture type and actual soft tissue conditions. Several types of osteosynthesis such as tension band wiring and its different modifications (K-wires, figure-of-eight wiring, equatorial-cerclage) were performed. Also locking plate fixation was done after its introduction to our trauma department in the year 2015.

**Fig. 1:**
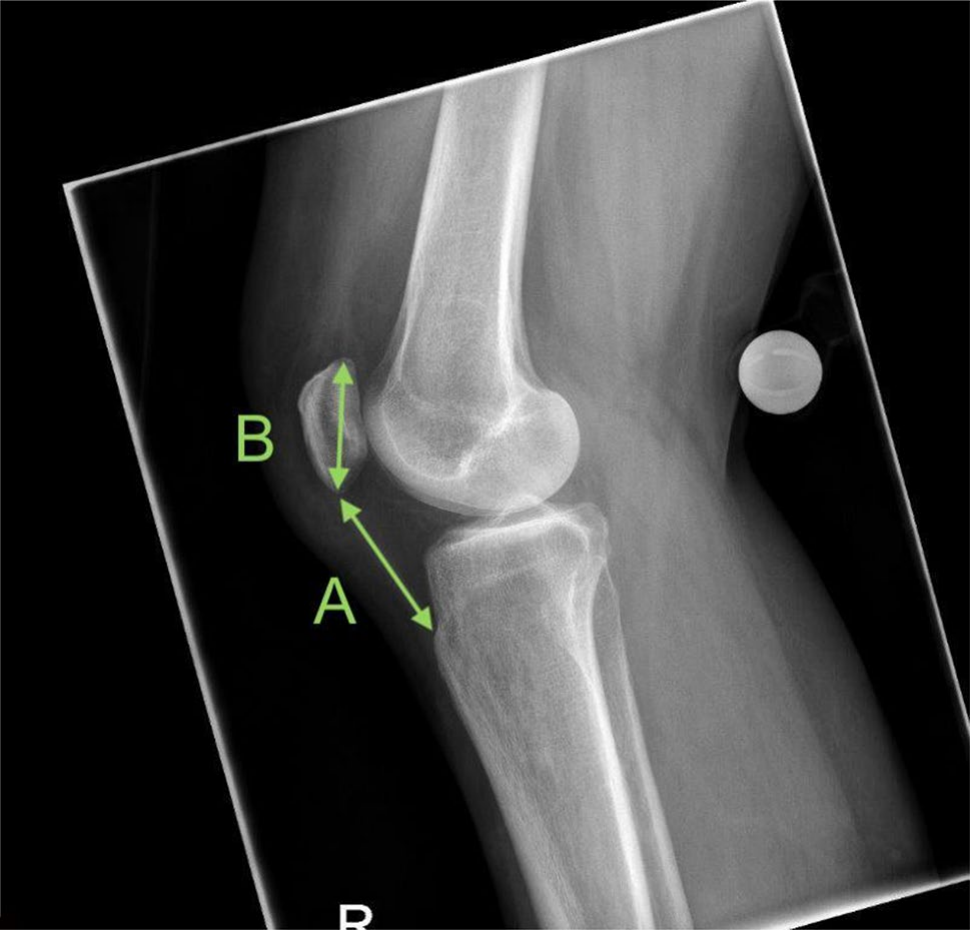
Lateral view (X-ray) of the knee showing the assessment of the Insall–Salvati Ratio (ISR, A/B)

Patella height was measured using the Insall–Salvati (IS) score on X-rays in lateral view. The Insall–Salvati Ratio (ISR) was assessed by measuring the distance from the tibial tuberosity to the inferior pole of the patella as well as by measuring the longest diameter of the patella (see Fig. 1). The ISR was assessed on X-rays of all enrolled patients primarily 2 days after surgery (group A) and secondarily at the latest follow-up appointment (group B). The indication for X-rays at the latest follow-up was made upon the department’s clinical standardized aftercare protocol (e.g., check for bone consolidation after 6 weeks, new complaints after consolidation or before implant removal). ISR-values between 0.8 and 1.2 were considered as normal, whereas in case of an ISR < 0.8 a patella baja was described and correspondingly a patella alta was diagnosed if the ISR was > 1.2 [14, 15]. The Chi-square test was used to compare the ISR of group A and B for statistical significance.

The second part of the presented work was the analysis of the functional outcome using a standardized self-assessment questionnaire (Munich Knee Questionnaire (MKQ)) [16]. The MKQ as a self-assessment test combines well elaborated clinical tests for knee injuries (Knee Injury and Osteoarthritis Outcome Score (KOOS), International Knee Documentation Committee (IKDC), Western Ontario Meniscal Evaluation Tool (WOMET), Lysholm Score and Tegner activity score) [16]. In this context the functional outcome was analysed in refer to the patella height and the fracture type. The Kruskal–Wallis test was used to compare the MKQ of different patella heights in terms of patella baja, norma or alta. The Mann–Whitney test was used for non-parametric testing of significant difference in functional outcome in AO classification of AO.34 type B and C fractures. Data are provided as arithmetic mean and standard deviation. D’Agostino & Pearson test showed a normal distribution of values. Statistical analysis was performed using the Sigma Stat 3.1 software (Systat Inc, Chicago, IL, USA) with a level of significance below 0.05 (*p* < 0.05).

## Results

The questionnaire was sent to 375 patients with patella fractures who were treated in our Level-I trauma center between the year 2003 and 2016. Overall 111 questionnaires were returned corresponding to a respond rate of 29.6%. Following the inclusion criteria, 54 patients (20 male, 34 female) with a mean age of 63.2 years were enrolled. The remaining 57 patients were excluded because of either incomplete questionnaires (*n* = 13), conservative treatment (*n* = 21), no and shorter follow-up with X-ray control (*n* = 18), respectively, or “MQK” follow up under 6 months (*n*= 5).

The enrolled patients presented with 10% AO.34 type B and 90% AO.34 type C fractures according to the AO classification. 50% of the cases were treated with tension band wiring, in 20% screw fixations, in 13.3% tension band wiring with screw fixation and in 6.6% locking plate fixation (Starplate®, Arthrex) was performed. In 58.1% of the patients implant removal was performed due to either symptomatic irritation by the implant or prominence of the implant. Eight patients suffered from postoperative complaints in terms of short follow-up implant failure so that revision surgery needed to be performed. Five of these cases were treated with tension band wiring, one patient suffered from a dislocation of the inferior patella pole after locking plate fixation and one patient received revisional surgery due to a plica syndrome along with implant failure of screw fixation.

Regarding the evaluation of ISR in group B the time point for the latest follow-up was set at a minimum of 6-week post-surgery. The follow-up time between group A and B accounted for a mean of 503.8 ± 655.7 days. The analysis of the ISR on lateral radiographs of the knee 2 days after surgery (Group A) showed 9.1% patella baja and 27.3% patella alta. The ISR assessed on the last follow-up (group B) revealed in 20.0% cases a patella baja and in 14.5% a patella alta. Proportionally the ratio of patella baja changed from group A to group B from 1:11 to 1:5, respectively, of patella alta from 1:3.6–1:6.9 (*p* = 0.05) (see Table 1). The mean ISR of patella baja, norma and alta of both groups showed no significant difference (*p* = 0.3) (see Table 2).

**Table 1:** Demonstration of patella baja, norma and alta in group A and B and overall functional outcome of MKQ in patella baja, alta and norma

	Patella baja (*n* = 16)	Patella alta (*n* = 23)	Patella norma	*P*
Group A	5*	15*	30	n.s.*
Group B	11*	8*	36	n.s.*
“MKQ”	65.4% ± 20.8	69.0% ± 18.2	61.8% ± 13.5	0.2

**Table 2:** Mean ISR of group A and group B in patella baja, alta and norma

Mean ISR	Patella baja	Patella alta	Patella norma	*P*
Group A	0.74*	1.36*	1.01	n.s.*
Group B	0.73*	1.39*	0.99	n.s.*

The evaluation of the MKQ was considered at a time frame from more than 6 months. The mean functional outcome revealed relatively good general results with a mean of 65.1% ± 14.9 in the MKQ. In detail, patients with an AO.34 type B presented a mean MKQ score of 74.5% ± 11.0 and AO.34 type C fractures with a mean of 64.0% ± 15.0. There was no significant different functional outcome assessed by the MKQ in patients with type B or C fractures (*p* = 0.1).

The functional outcome according to the MKQ accounted for patients with patella baja for 67.1% ± 17.9 and for patella alta for 69.0% ± 18.2. The overall MKQ Score with normal ISR was 62.2% ± 14.1.

**Fig. 2:**
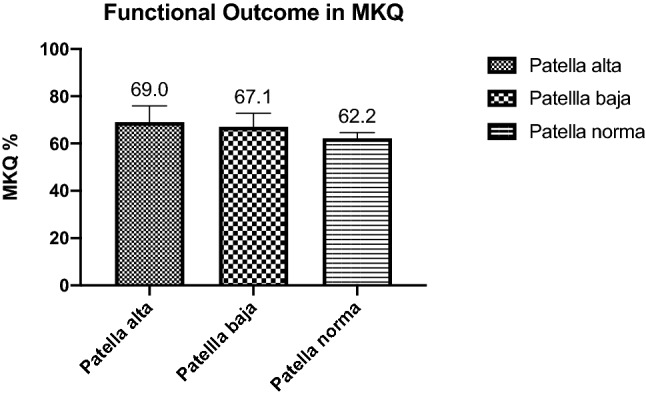
Functional outcome in the mean MKQ score assessed on the latest follow-up is shown for all patella heights: patella baja, alta and norma (*p* = 0.2)

No significant difference in functional outcome in the MKQ resulted comparing patella baja, norma and alta (*p* = 0.9, see Fig. 2).

## Discussion

The purpose of this retrospective study was to evaluate the change in patella height and functional outcome in patella fractures. Generally speaking several surgical treatment options are available according to the fracture type using the AO classification. Open or closed fixation using tension band wiring with its modifications as well established techniques for transverse fractures of the patella are widely used [17–19]. Dy et al. reported in their meta-analysis about several complications of tension band wiring resulting in a less functional outcome and high revision rates in up to one-third of all patients including loss of reduction and secondary dislocation, implant failure or implant discomfort [20, 21]. There are several studies in the actual literature on the functional outcome of the knee joint following patella fracture. In this context Wurm et al. focused on the locking plate fixation treatment [22–25] commonly postulating good results for locking plate fixation in the treatment of comminuted patella fractures. Furthermore, Ellwein et al. reported about restored function of the knee within 6 months after surgery [24]. To the best of our knowledge the presented study shows for the first time results on the functional outcome in refer to the patella height following surgically treated patella fractures. Our results demonstrate that different postoperative patella heights apparently do not influence the functional outcome.

In general especially a patella baja position is conjoined with more stress related complaints, since the lower height of the patella can lead to structural changes of the patellar tendon and functional weakness of the quadrizeps muscle correspondingly [26]. In addition, congenital soft tissue disorders, posttraumatic or postoperative changes of the soft tissue due to scaring and tissue adherence are supposed to play a key roll in anterior knee pain syndromes [27, 28]. In the literature an infra-patellar contracture syndrome or premature osteoarthritis due to patellofemoral misalignment was already well described decades ago but still undergoes intensive research [29, 30]. Also the surgical approach might simultaneously result in a patella baja and its corresponding complications in terms of either impingement or a lower lever arm along with decreased range of motion [31]. In this context our results show a proportional decrease of patella height, since more cases of patella baja and less patella alta were found in total at the latest follow-up. In our opinion this might be triggered by evolution of adhesive or scar tissue after surgery. Another reason may be the postoperative rehabilitation program including a limited range of motion of the knee joint for several weeks. However, so far, the radiological finding of a patella baja was not clinically proven in changes of the functional outcome, but the presented results of our study with a relatively short follow-up time show that patella height does not influence postoperative functional outcome.

For the retrospective assessment of the functional outcome following patella fracture the well-known Munich Knee Questionnaire (MKQ) was used. The MKQ presents a self-assessment test allowing for a subjective and objective patient-based evaluation of post-therapeutic problems of the knee. In the presented study, patients suffering from either a postoperative patella alta or baja showed good to relatively good functional outcome in the MKQ. The functional outcome showed no significant difference between patients with patella baja, norma and alta. Moreover, no noticeable difference in the functional outcome was found in relation to the severity of the patella fracture according to the AO classification. In the current literature corresponding studies reveal good results in the SF-36/KOOS Score and Special Surgery Knee Scoring (SSKS) after surgical treatment of patella fractures [32] [33, 34], so it can be concluded that the recent literature reflects the presented results of the MKQ after surgical therapy of patella fractures.

The actual study presents with several limitations. First of all due to the retrospective study design a selection bias was accepted. The high dropout rate of all screened patients, who were treated for patella fractures between 2003 and 2016 may alter the results of functional outcome. This circumstance may influence the functional outcome proportionally, as patients with bad functional outcome may have not been considered in the general results. A subgroup analysis of functional outcome in different surgical technique was not performed, as the number of patients per performed procedure was too low in total to achieve statistically reliable results. This may have influenced the functional outcome of different fracture types. The high drop-out rate of our initially screened 375 patients is mainly explained by the restricted answer rate (< 30%) of the MKQ along with the inclusion criteria of a minimum follow-up for the ISR change and MKQ. Furthermore, only postoperative changes were evaluated. We consider the high drop-out rate of the initially screened patients as a problem of inconsistent patients’ answer in this long observation interval and restricted utilization of incorrect MKQs. This sampling bias may influence the results by not including bad functional outcomes of these patients. We accept this selection bias as a concern in our results.

Due to the retrospective design of the presented study but also due to radiation exposure reasons the patella height of the contralateral, healthy knee joint was not considered. This may have restricted the incidence of a preexisting patella baja or alta and may have influenced the results of postoperative change in patella height.

In addition the authors have to admit that the evaluation of the patella height differs significantly between the different scoring systems. The interobserver reliability on plain lateral radiographs is low; therefore, we used the ISR as a well established method without high interobserver differences, to discriminate between patella baja, norma and alta [35]. But this in fact, may also influence the incidence of patella baja or alta in our patient cohort.

## Conclusion

Restoring the extensor mechanism of the knee joint still remains crucial in treatment of patella fractures. In this context the common literature believes that different levels of patella height have an influence on anterior knee pain. According to our results, the patella height does not significantly affect the functional outcome following surgical fracture treatment. In addition, our data demonstrate well that there is also no significant difference of the functional outcome referring to the severity of the patella fracture using the AO classification.

## Abbreviations

Patella baja: Inferior patella height
Patella alta: Elevated patella height
Patella norma: Normal patella height
MKQ: Munich Knee Questionnaire
IS: Insall–Salvati
ISR: Insall–Salvati Ratio
n.s.: Not significant
